# Intraspecific differences in the photosynthetic responses to chloroplast CO_2_ and photon flux density at different leaf temperatures of four grapevine cultivars grown in common outdoor conditions

**DOI:** 10.1002/pld3.595

**Published:** 2024-06-05

**Authors:** Dennis H. Greer

**Affiliations:** ^1^ National Wine and Grape Industry Centre, School of Agricultural and Wine Science Charles Sturt University Wagga Wagga New South Wales Australia

**Keywords:** *A/ci* responses, grapevines, heat tolerance, intraspecific variation, leaf temperature, light responses

## Abstract

Comparative measurements of four *Vitis vinifera* cultivars were undertaken to assess assimilation tolerance to the high growth temperatures currently pervading Australian and other wine growing regions. The cultivars, cvs. Chardonnay, Merlot, Semillon, and Shiraz, were all grown in common growth conditions, and an hypothesis promulgated genotypic variation in assimilation and in the leaf temperature dependency. Assimilation responses to varying light intensity and to varying chloroplast CO_2_ at a range of leaf temperatures (15–45°C) were measured in leaves of each cultivar in mid‐summer. Light response curves revealed marked genotype differences in maximum assimilation, but temperature effects also varied. Semillon leaves were most sensitive to temperature, with marked and steep differences in assimilation at different temperatures while Chardonnay and Merlot were least sensitive, with relatively flat responses. There were also marked cultivar differences in response to CO_2_ and significant effects of leaf temperature. CO_2_‐saturated assimilation varied markedly, with Semillon and Merlot leaves most responsive to temperature, although there were differences in optimum temperatures and maximum rates. Chardonnay leaves remained least tolerant, with lowest rates of assimilation across most temperatures. Assimilation at 45°C also separated the cultivars and two cultivars had higher rates than at 15°C while Chardonnay and Merlot leaves had higher rates at 15°C. There were no cultivar differences in the temperature dependency of Ribulose 1,5‐bisphosphate (RuBP) carboxylation, but Semillon had a much steeper temperature dependency on RuBP regeneration than the other cultivars. All these responses confirmed the hypothesis and concluded the high‐temperature tolerance of Semillon and Shiraz and the poor adaptability of Chardonnay and possibly Merlot to perform in the current high‐temperature growth conditions.

## 1 INTRODUCTION

Grapevines (*Vitis vinifera*) are of enormous economic importance to Australia, and many other hot places, but are being grown in ever increasing high‐temperature conditions, especially experiencing heat stress (Webb et al., 2009). For example, in the decade between 2000 and 2010, it was uncommon for Australian summer temperatures to exceed 40°C for more than a few days (Greer & Weedon, 2013); however, in the summer of 2009, a +40°C heat event lasted for 14 days. While the decade 2010–2020 has not been comparably assessed, during both the 2012/2013 and 2013/2014 growing seasons, 10 and 21 days of above 40°C maximum temperatures occurred (Abeysinghe et al., 2019). Thus, in many vineyards in regional Australia, vines are routinely growing and developing in temperatures at least between 40 and 45°C for a part of the growing season, now known to occur as early as November (flowering) and as late as February (harvest). Coupled with the high radiant load during the summer (Greer & Weedon, 2012a), these high‐temperature events are deleterious to vine productivity (Abeysinghe et al., 2019; Caravia et al., 2016; Greer & Weston, 2010a). Evidence that the climate is shifting (Jones & Davis, 2000; Sadras & Petrie, 2011) suggests these high‐temperature events will also continue to increase in frequency and intensity and understanding which grapevine cultivars will perform well under these conditions will be necessary to adjust productivity to this changing climate.

Across the *V. vinifera* species, there is a vast number of cultivars, having been cultivated for well over 5000 years, and cultivars have originated by chance cross fertilization, natural selection, and direct crosses with other species (Iland et al., 2011). There are at least 70 cultivars of relevance to Australian vineyards (Kerridge & Antcliff, 1996). Despite this large array of intraspecific variation, relatively little knowledge is known of most of the *V. vinifera* cultivars and how well they are matched, not just with the current climate, but how suitable they will perform in a changing climate. The knowledge of understanding the physiological and environmental responsiveness attributes of this vast species is, therefore, relatively poor. To date, comparative studies of two cultivars were relatively common, including cvs. Chardonnay and Airén (Gómez‐del‐Campo et al., 2002), cvs. Manto Negro and Tempranillo (Escalona et al., 2003), cv. Montepulciano and Sangiovese (Palliotti et al., 2009), and cvs. Tinta Amerela and Periquita (Chaves et al., 1987). By contrast, in a seminal study, Rogiers et al. (2009) compared the gas exchange of 10 commercially grown *V. vinifera* cultivars, all growing in a common field site and demonstrated marked genotypic variation in light‐saturated photosynthesis, stomatal conductance, stomatal density, and transpiration. Additionally, Costa et al. (2012) compared five field‐grown grapevine cultivars (cvs. Touriga Nacional, Aragonez, Syrah [aka Shiraz], Trincadeira, and Cabernet Sauvignon) and maximum rates ranged between 5.6 μmol m^−2^ s^−1^ for Syrah and 12.3 μmol m^−2^ s^−1^ for Touriga Nacional (Costa et al., 2012). However, the effects of high temperatures on the gas exchange attributes of these various cultivars have not been well addressed. Again, although gas exchange was not measured, Greer and Weston (2016) measured canopy temperatures of four common cultivars, cvs. Pinot Noir, Sauvignon Blanc, Shiraz, and Riesling vines, all growing in a common field environment and were exposed to a major heat event during the growing season. The results indicated that genotypic variation occurred in canopy temperature, where western canopies of cv. Sauvignon Blanc vines reached 45°C when air temperature reached 39°C whereas cv. Riesling vines remained below 40°C at the same time while canopy temperatures of cvs. Pinot Noir and Shiraz vines also exceeded 40°C, by 2–3°C. It remained uncertain what attributes might have conferred these cultivar differences in heat sensitivity/tolerance. However, stomatal conductance has been shown to vary between these same cultivars (Rogiers et al., 2009), for example, cv. Pinot Noir vines had the highest conductance (376 mmol m^−2^ s^−1^) and hence transpiration and cv. Shiraz vines had the lowest (215 mmol m^−2^ s^−1^) with Riesling vines intermediate, and therefore, these stomatal conductances and transpiration rates did not reflect the inherent heat sensitivity of these cultivars. Hence, there are clear indications of inherent genotypic variation in gas exchange attributes and heat stress between some grapevine cultivars.

Greer and colleagues (Greer et al., 2011; Greer & Sicard, 2009; Greer & Weedon, 2012b) have comprehensively investigated the heat tolerance of the economically important grapevine cultivar cv. Semillon growing both in commercial vineyards and in controlled environments. Flowering, berry ripening, and sugar accumulation were especially sensitive phenological stages to temperatures above 40°C (Greer & Weston, 2010a) but physiologically growth, photosynthesis, stomatal conductance, and Ribulose 1,5 carboxylation (RuBP) carboxylation and regeneration were all maximal at 30–35°C and, although compromised at 40°C, were not deleteriously affected (Greer & Weston, 2010b). Notably, photosynthesis along the Semillon shoots prior to and after the 2009 heat wave caused a 60% reduction in photosynthesis but a 55% increase in stomatal conductance and 130% increase in transpiration (Greer & Weedon, 2017), clearly these attributes were an adaptation of this cultivar to the heat event. Furthermore, Greer (2012) has established that leaves of Semillon vines grown in the vineyard were fully capable of photosynthesis at 45°C with only a 30% reduction from the maximum rates at 25–30°C. Similarly, the cultivar Sultana was also capable of photosynthesis at 45°C although reduced by about 80% of the maximum rate at 30°C (Kriedemann, 1968). For the cultivar Thompson Seedless, however, photosynthetic rates at 40°C were about 50% of the maximum whereas at 45°C, the rates were negative (Mullins et al., 1992); thus, this cultivar clearly lacked high‐temperature tolerance.

On an individual cultivar basis, several other studies have assessed the impacts of high temperatures on gas exchange and other attributes. For example, Soar et al. (2009) exposed irrigated field‐grown cv. Shiraz vines to 40°C and reported little impact on the photosynthetic rates. For the same cultivar, by contrast, exposure of vines to temperatures between 40 and 43°C (Caravia et al., 2016) caused about 60% inhibition of photosynthesis. However, when measured briefly (within minutes) to 45°C at selected stages throughout the growing season (Greer, 2020), leaves of outdoor‐grown cv. Shiraz vines maintained light‐saturated photosynthetic rates at 5–10 μmol m^−2^ s^−1^. When measured at 30°C, rates averaged 10–15 μmol m^−2^ s^−1^, thus indicating relatively high tolerance to these supra‐high temperatures. It remains uncertain how growth conditions in each of these studies on the same cultivar may have influenced the different responses. Furthermore, differences in exposure times may account for some differences between these studies, although in Greer's (2020) study, the vines were exposed to 13 days of above 40°C air temperatures including 2 days at 45°C during the growing season, which might partially explain the results. By contrast, during a growing season where air temperatures remained between 35 and 40°C, both cvs. Chardonnay and Merlot vines maintained light‐saturated photosynthetic rates between 5 and 8 μmol m^−2^ s^−1^, at a leaf temperature of 45°C (Greer, 2018a), thus largely in keeping with those rates for Shiraz vines growing in similar conditions. Chardonnay vines grown in Burgundy, France, had light‐saturated photosynthetic rates of 12 μmol m^−2^ s^−1^ when measured at 27°C (Gómez‐del‐Campo et al., 2002) and comparable with rates in the study above. As another example of the genotypic variation, the cultivar Trebbiano Toscano grown in controlled conditions maintained light‐saturated rates at 27°C growth temperature of 4–6 μmol m^−2^ s^−1^ over 100 days of growth whereas at 35°C growth temperature, photosynthetic rates ranged between 1 and 2 μmol m^−2^ s^−1^ (Ferrini et al., 1995), suggesting inherently low high‐temperature tolerance of this cultivar. It remains uncertain, however, if the high‐temperature tolerance of grapevine cultivars was dependent on the actual growth conditions or inherent genotypic effects.

Accordingly, an hypothesis is advanced that grapevine cultivars will express inherent genotypic differences in gas exchange when challenged with high temperatures. To evaluate this hypothesis, the objective was to directly compare the gas exchange and photosynthetic attributes of four important grapevine cultivars in relation to leaf temperature and to ascertain if all cultivars responded equally to increasing leaf temperatures. The cultivars included Semillon, originating in Bordeaux (Mullins et al., 1992); Chardonnay, originating in Burgundy; Merlot, originating in Bordeaux; and Shiraz, originating in the Rhône Valley, France, although grown in Australian vineyards for at least 70 years. In all cases, the potted vines were grown in identical conditions. These is some evidence that grapevines growing in high‐temperature conditions bias their photosynthetic temperature response towards high temperatures, and this will be examined across the four cultivars.

## 2 MATERIALS AND METHODS

### 2.1 Plant material and growth conditions

This study was undertaken at the Charles Sturt University National Wine and Grape Industry plant growth facilities in the Riverina, New South Wales, Australia, during the mid‐summer (January–February) of the 2018/2019 growing season. For the experimental plan, eight potted 8–10‐year‐old vines of *V. vinifera* cv. Semillon, cv. Chardonnay, cv. Merlot, and cv. Shiraz and all grown on own roots were grown each in 30–52 L pots containing a commercial bulk composted potting mix with liquid fertilizer (Megamix Plus, Rutec, Tamworth, Australia). Some additional applications of sulfate of ammonia as a source of nitrogen (Richgro Garden products, Jandakot, WA, Australia) were made at a rate of 100 g at flowering time. These vines were grown in rows with 3 m spacing between rows and 1 m spacing between vines. The fruiting vines were grown in a wire framed bird exclusion cage but otherwise were exposed to the natural growth conditions. For all cultivars, the vines were drip irrigated, and the pots were irrigated three times daily at 10 min duration, delivering 2.4 L h^−1^. Budbreak for all cultivars occurred in mid‐September, full bloom occurred in early November, and harvest occurred in early to mid‐February. This site was characterized by long‐term average maximum air temperatures of 29.4, 31.7, and 30.8°C during the summer period (December to February) but also subjected to afternoon temperatures often exceeding 40°C for several hours over several days to weeks (Abeysinghe et al., 2019; Greer & Weedon, 2012b).

### 2.2 Photosynthetic response to photon flux density

To determine the photosynthetic response of the leaves of each cultivar to photon flux densities (PFD) at different leaf temperatures, a fully expanded leaf was attached to the leaf chamber of the LI6400 open gas exchange system (LiCor BioSciences, Lincoln, NE, USA) with the LI6400‐40 leaf chamber fluorometer light source. In each case, the leaf was initially exposed to a high PFD (~1500 μmol (photons) m^−2^ s^−1^) and a constant leaf temperature, and when the photosynthetic rates were steady, the PFD was progressively decreased in selected steps to about ~1 μmol (photons) m^−2^ s^−1^ (dark). The leaf temperatures during each light response were held constant and ranged from 15 to 45°C; however, on occasions because of the prevailing ambient conditions, it was not possible to achieve this whole range of leaf temperatures, especially the highest temperature. The CO_2_ concentration was controlled throughout at 400 μmol mol^−1^, and vapor pressure was maintained through a simple humidifying system (Greer, 2018c). For each temperature and cultivar, the light responses were repeated four to five times, taking 20 days to complete measurements and a new leaf was used in all cases.

### 2.3 Photosynthetic response to chloroplast CO_2_

Measurements of the photosynthetic response to intercellular CO_2_ at the selected leaf temperatures (15–45°C) were undertaken on youngest fully expanded leaves of each cultivar using the LI6400 open gas exchange system. On each occasion, prior to measurement (Sharkey, 2016), the leaf chamber was checked for the seals and replaced if required to ensure no leakage occurred at low CO_2_ concentrations. For all and every *A/c*_*i*_ response, a new fully expanded leaf was used for all cultivars and all occasions. All responses were conducted at constant leaf temperatures, nominally between 15 and 45°C, but again not all the low and high temperatures could be achieved with the LI6400 Peltier temperature control system because of prevailing ambient conditions on some days. For each *A/c*_*i*_ response, the procedure commenced with an initial CO_2_ concentration of 400 μmol mol^−1^ and when rates were steady, the CO_2_ was reduced in selected steps to 30–50 μmol mol^−1^ before returning the CO_2_ to 400 μmol mol^−1^ to ensure photosynthesis had returned to the same rate. Then, the CO_2_ concentration was increased in steps of 100–200 μmol mol^−1^ to reach a maximum concentration of about 1800 μmol mol^−1^. For all cultivars and on all days, leaf temperatures were progressively increased to the set points in accordance with the rising air temperatures throughout the day to ensure the leaves were well acclimated to each cuvette temperature. In all cases, the PFD during each *A/c*_*i*_ response was set at 1400 μmol (photons) m^−2^ s^−1^ for the measurements but in all cases was saturating (Greer, 2017). The vapor pressure was not controlled but managed as stated earlier. Each *A/c*_*i*_ response was repeated four to five times for each cultivar and leaf temperature, and all measurements occurred over 20 days during the summer growing season.

The LI6400‐40 leaf chamber fluorometer enabled simultaneous chlorophyll fluorescence measured at each CO_2_ step and the steady‐state fluorescence in the light (*F*_s_ *'*) and the maximal fluorescence in the light (*F*_m_ *′*).

### 2.4 Data analysis

All data were analyzed assuming a fully randomized experimental plan was adopted, and the data were analyzed using a general linear model (GLM) procedure using SAS 9.3 (SAS Institute Inc., Cary, NC, USA), and least squares means and standard errors were calculated. The GLM model included cultivar and temperature as main effects and cultivar × temperature as the interactive effect. The *P* (probability of GLM model significance) and *r* ^2^ (% variation accounted by model) was determined as a fitness of the model and was applied to all photosynthetic attributes tested by the GLM procedure.

The light response data were analyzed by fitting a hyperbolic (*Tanh*) tangent function to each light response according to Greer and Halligan (2001). The procedure involved the use of non‐linear regression analysis in SAS to fit the function and determine the light‐saturated rates of photosynthesis at ambient CO_2_ (*A*_400_), apparent photon yields (ϕ_a_), and rates of dark respiration (*R*_*dark*_). The PFD at which photosynthesis was fully light saturated (*PFD*_*sat*_) was determined as *Atanh* (.99) * *A*_*max*_/ϕ_a_ where *Atanh* is the inverse of *Tanh* and set at 99% saturation in all cases.

The fitting of the Farquhar et al. (1980) C_3_ model of photosynthesis to the *A/c*_*i*_ data followed the procedure of Greer and Weedon (2012b) using non‐linear regression in SAS. The internal CO_2_ concentrations (*c*_*i*_) were first converted to chloroplast concentrations (*c*_*c*_) using the corrected (according to Greer, 2022; van der Putten et al., 2018) electron transport rate determined by the simultaneous chlorophyll a fluorescence and gas exchange measurements to estimate mesophyll conductance (*g*_*m*_) according to Flexas et al. (2007) and Pons et al. (2009). Subsequently, the maximum rate of Ribulose 1,5‐bisphosphate (RuBP) carboxylation (*V*_*cmax*_) and the maximum rate of RuBP regeneration (*J*_*max*_) were calculated with SAS using the temperature‐dependent coefficients for the Michaelis constants of CO_2_, oxygen, and the CO_2_ compensation point in the absence of mitochondrial respiration from Sharkey (2016). To determine the temperature dependency of *V*_*cmax*_ and *J*_*max*_ to temperature, the approach adopted by Medlyn, Dreyer, et al. (2002) and Greer and Weedon (2012a) was followed using the in vivo temperature dependencies as determined by Bernacchi et al. (2001). These data were analyzed statistically using the GLM procedure to determine the interaction between the cultivars and leaf temperatures.

## 3 RESULTS

### 3.1 Photosynthetic PFD responses

**FIGURE 1:**
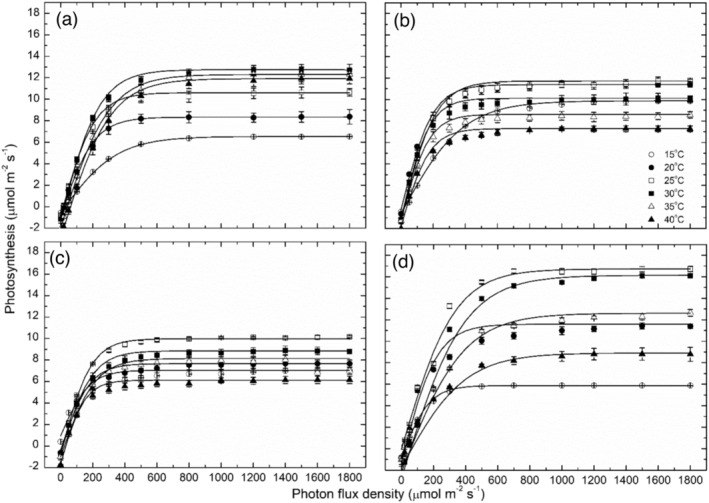
Photosynthetic responses to PFD (mean ± SE, *N* = 4–5) at a range of leaf temperatures as indicated for the four grapevine cultivars: (a) Shiraz, (b) Merlot, (c) Chardonnay, and (d) Semillon. Because there was limited data at 45°C, additional data from the other studies enabled measurements at this temperature to be included in the analysis.

The photosynthetic responses to increasing PFDs for the four grapevine cultivars are shown in Figure 1. In each case, the responses all fitted the hyperbolic tangent function (Greer & Halligan, 2001) well (*r* ^2^ = .95–.98; *P* < .001) and were fully saturated at high PFDs. For most cultivars, the sensitivity of the PFD responses to leaf temperature was somewhat subdued. Light‐saturated rates ranged in Chardonnay leaves from 6.2 ± .35 at 40°C to 10.1 ± .36 μmol m^−2^ s^−1^ at 25°C (1.6‐fold) whereas in Semillon leaves, light‐saturated photosynthetic rates ranged from 8.8 ± .45 at 40°C to 16.7 ± .25 μmol m^−2^ s^−1^ at 25°C (1.9‐fold). The remaining cultivars fitted in between these two ranges, notably Merlot leaves were maximal at 11.8 ± .12 μmol m^−2^ s^−1^ at 25°C (1.7‐fold) while Shiraz leaves were maximal at 12.7 ± .25 μmol m^−2^ s^−1^ at 30°C (1.6‐fold). Thus, the maximum light‐saturated photosynthetic rates for each cultivar were significantly (*P* < .01) and inherently different.

**FIGURE 2:**
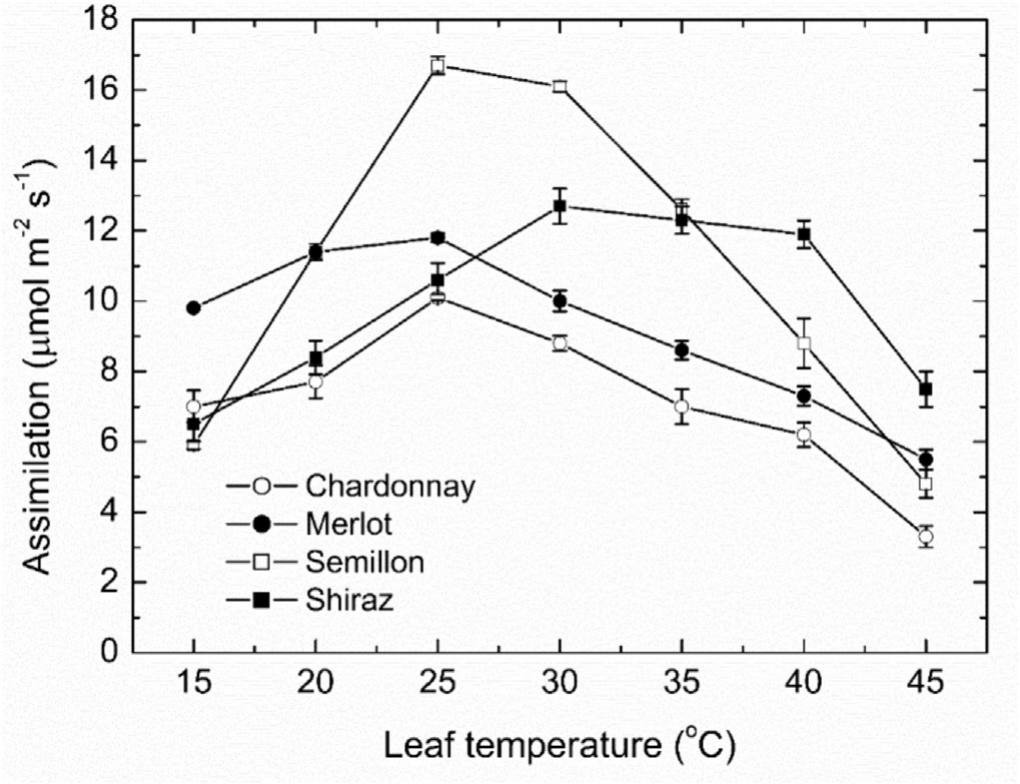
Light‐saturated photosynthetic rates (mean ± SE, *N* = 4–5) at ambient CO_2_ concentration as a function of leaf temperature for four grapevine cultivars as indicated. Additional data on new leaves were included with the photosynthetic light response data especially at the highest temperatures for completeness. These measurements were conducted at a PFD of 1400 μmol (photons) m^−2^ s^−1^ and at a CO_2_ concentration of 400 μmol mol^−1^.

The overall assimilation responses to leaf temperature (Figure 2) demonstrated clearly that the light‐saturated maximum photosynthetic rates were highly sensitive to leaf temperature; however, there were clear cultivar differences; and the interactions between cultivar and temperature were highly significant (GLM analysis; *P* < .01, *r* ^2^ = .78). Both Chardonnay and Merlot leaves were moderately sensitive to temperature between 15 and 30°C, with relatively small differences in assimilation mostly between 6 and 11 μmol m^−2^ s^−1^ and broadly optimal between 20 and 25°C, with consistent differences in assimilation between 15 and 30°C. However, from 25 to 45°C, the photosynthetic rates declined steeply and about linearly, to significant differences at 45°C, 3.3 ± .3 μmol m^−2^ s^−1^ for the Chardonnay leaves and 5.5 ± .3 μmol m^−2^ s^−1^ for the Merlot leaves. Hence, both cultivars were clearly sensitive to the increasing leaf temperatures. By comparison, photosynthetic rates for the Shiraz leaves increased markedly between 15 and 30°C from 6 to 13 μmol m^−2^ s^−1^, hence highly temperature sensitive, and only declined slightly at the higher temperatures, averaging 12.7 ± .5 μmol m^−2^ s^−1^ at 30°C. Notably, photosynthesis was broadly optimal at 30–35°C. However, photosynthetic rates of the Semillon leaves increased most steeply to a sharp optimum at 25°C, averaging 16.7 ± .5 μmol m^−2^ s^−1^, and therefore, markedly higher than all other cultivars. Thereafter, the assimilation rates declined also most steeply but above 40°C, they were within the range of the other cultivars. In all cases, however, the decreases in photosynthesis between 35 and 45°C averaged 38% to 47% lower rates for Chardonnay and Semillon, but for Merlot and Shiraz vines, the decrease averaged 60% to 64%.

The assimilation rates at the lowest leaf temperature (15°C) for Semillon and Chardonnay leaves ranged between 5.9 and 7.0 ± .4 μmol m^−2^ s^−1^, and the rates for Shiraz were well within this range. However, for the Merlot leaves, the rates at this low temperature were significantly (cultivar effect: *P* < .01) highest at 9.8 ± .4 μmol m^−2^ s^−1^, and these cultivar differences were somewhat retained at 20°C. Hence, at the low‐temperature range, cultivar differences were apparent but not well discriminated.

At the high‐temperature range (40–45°C), there were clear and significant differences in assimilation rates between the cultivars, where Chardonnay averaged 3.3 ± .23 μmol m^−2^ s^−1^ at 45°C while the Merlot leaves averaged 5.5 ± .18 μmol m^−2^ s^−1^. By contrast, the assimilation rates of the Shiraz leaves were significantly (*P* < .01) highest at 7.5 ± .33 μmol m^−2^ s^−1^, thus, clearly the most tolerant at the higher temperatures. Rates for the Semillon leaves were comparable with those for Merlot. It was notable that for cvs. Chardonnay and Merlot that the rates at 45°C were all lower than those at 15°C while for cv. Semillon, the same occurred but the difference was smaller. Only Shiraz vines had higher rates at 45°C than at 15°C, but again, the difference was relatively small. It was evident, therefore, that the photosynthetic light responses between the cultivars were inherently different as to the pattern of response to temperature, the maximum assimilation rates, and the temperature that each was optimal for assimilation, explaining the statistical interaction between cultivar and leaf temperature.

**FIGURE 3:**
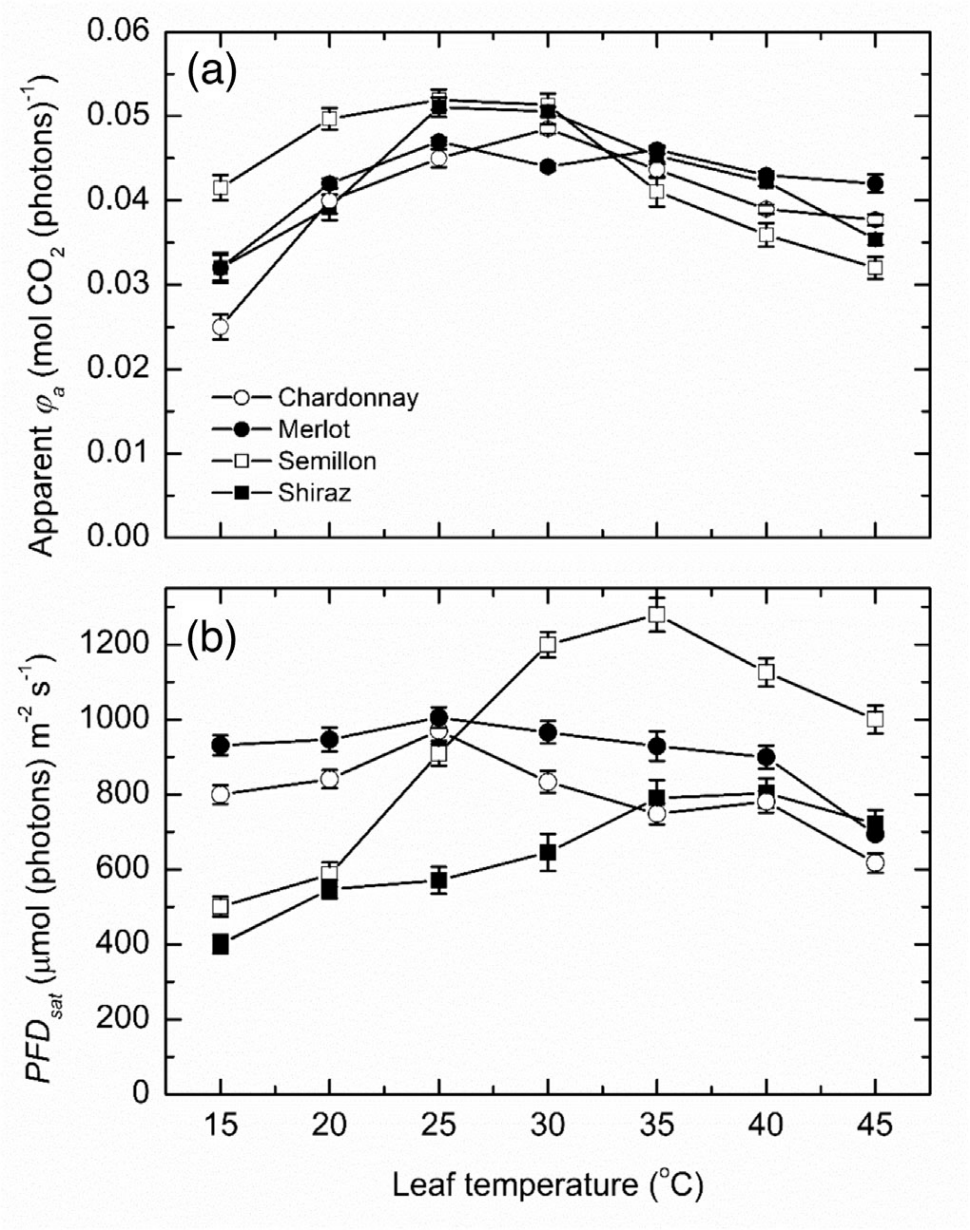
The photon yield of photosynthesis (mean ± SE, *N* = 4–5) as a function of leaf temperature (a) and the photon flux density at photosynthetic light saturation (b) for the four grapevine cultivars as indicated. These data were derived from the fitting of the hyperbolic tangent function as indicated in Figure 1.

Across the cultivars, there was a consistent pattern of temperature responses for the apparent (non‐saturated) photon yield (Figure 3a) indicative of a weak cultivar × temperature interaction but a strong cultivar effect (*P* < .01, *r* ^2^ = .69). For Chardonnay, Merlot, and Shiraz vines, the photon yields increased relatively steeply between 15 and 25°C, and maximum yields averaged between .049 and .055 ± .004 mol CO_2_ (photons)^−1^ for all three cultivars. Thereafter, the photon yields declined progressively, such that at 45°C, the photon yields averaged between .0354 and .0416 ± .004 mol CO_2_ (photons)^−1^, highest in Merlot and lowest in Shiraz. For Semillon, the photon yields increased less steeply between 15 and 25°C because the photon yield was significantly highest and between 15 and 20°C, averaged .0456 ± .005 mol CO_2_ (photons)^−1^ but also significantly higher at the optimum temperature, 25°C, at .054 ± .005 mol CO_2_ (photons)^−1^. By contrast, the photon yield for this cultivar declined most steeply with increasing leaf temperature such that at 45°C, the photon yield averaged .032 ± .0025 mol CO_2_ (photons)^−1^, hence significantly lower compared with the other cultivars. Therefore, while the overall responses to temperature were generally similar, there were marked cultivar differences in the photon yields especially at the lowest and highest temperature. Notably, Chardonnay vines had the lowest photon yield at 15°C, and Semillon had the highest.

For the PFDs saturating photosynthesis (*PFD*_*sat*_), there was a significant (*P* < .01; *r* ^2^ = .72) cultivar × temperature interaction. For both Chardonnay and Merlot, there was some evidence that leaf temperature had an effect (Figure 3b), with *PFD*_*sat*_ mostly between 400 and 800 ± 25 μmol (photons) m^−2^ s^−1^ across all leaf temperatures but with a slight indication of a maximum response at 25°C. However, for both Semillon and Shiraz, *PFD*_*sat*_ tended to increase about linearly from ~400 ± 15 μmol (photons) m^−2^ s^−1^ at the lowest temperature to maxima of 700–800 ± 35 μmol (photons) m^−2^ s^−1^ at 35–45°C for Shiraz, consistent with the other cultivars, and about 1200 ± 70 μmol (photons) m^−2^ s^−1^ at 35°C for Semillon; hence, there was some similarity in the temperature response pattern. However, these differences in patterns created marked cultivar differences in the saturating PFD; for Shiraz vines, this was mostly evident below 30°C where the lowest PFDs required to saturate photosynthesis occurred whereas both Chardonnay and Merlot required much higher PFDs to saturate photosynthesis at the low‐temperature range. Consistent with the highest rates of assimilation, Semillon vines also required much higher PFDs to saturate photosynthesis but increased very steeply between 20 and 35°C. Furthermore, *PFD*_*sat*_ of the Semillon leaves remained much higher than the other cultivars at 30–45°C. Thus, not only were the intraspecific differences in the PFDs saturating photosynthesis but there were also marked differences in the leaf temperature response.

### 3.2 Photosynthetic response to chloroplast CO_2_ concentration

**FIGURE 4:**
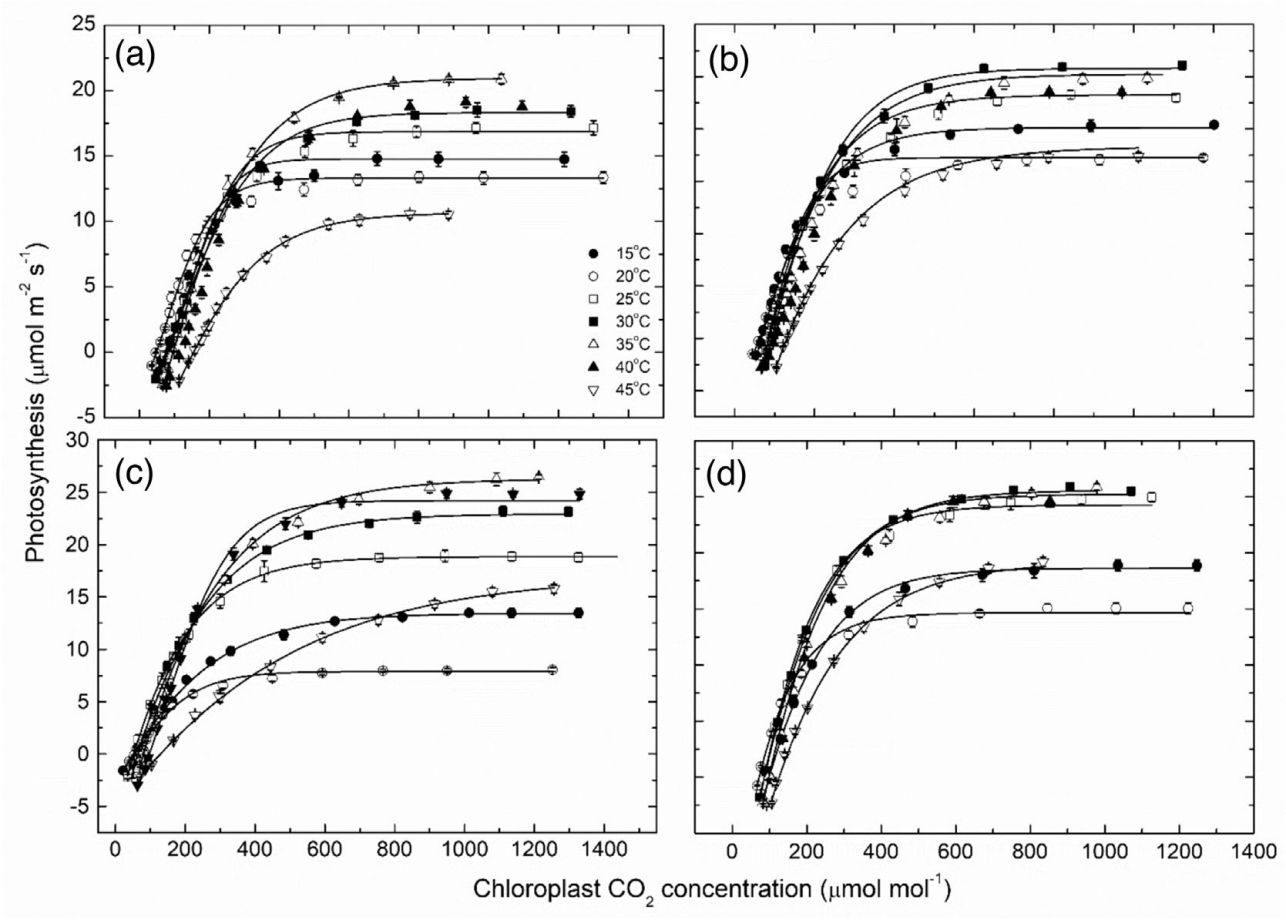
Photosynthesis as a function of chloroplast CO_2_ concentration (mean ± SE, *N* = 4–5) at a range of leaf temperatures as indicated for four grapevine cultivars: (a) Chardonnay, (b) Merlot, (c) Semillon, and (d) Shiraz. In all cases, the lines fitted to these data were from the C_3_ model of photosynthesis according to Farquhar et al. (1980). Note lines for the 40°C data in graphs (a), (b), and (d) have been excluded for clarity.

The photosynthetic responses to increasing chloroplast CO_2_ at leaf temperatures ranging between 15 and 45°C for the four grapevine cultivars are shown in Figure 4.

In each case, there was a strong curvilinear response to temperature, with minima between ranging between 8.1 ± .2 μmol m^−2^ s^−1^ at 15°C for Semillon and 1.5 ± .5 μmol m^−2^ s^−1^ at 45°C in Chardonnay leaves. Shiraz and Merlot vines both had their lowest CO_2_‐saturated rates at 15°C, between 15.0 ± .6 μmol m^−2^ s^−1^ in Shiraz leaves and 17.2 ± .4 μmol m^−2^ s^−1^ for Merlot vines. Maximum CO_2_‐saturated rates (*A*_*max*_) were typically at either 30 or 35°C and ranged from 20.9 ± .36 μmol m^−2^ s^−1^ in the Chardonnay leaves to 26.5 ± .38 μmol m^−2^ s^−1^ in the Semillon leaves, with Merlot and Shiraz slightly lower at 26.0 ± .6 μmol m^−2^ s^−1^. There were also marked cultivar differences in the photosynthetic rates at 45°C, with Chardonnay most compromised at 1.5 ± .5 μmol m^−2^ s^−1^ while the other three cultivars maintained rates between 17 and 19 ± .7 μmol m^−2^ s^−1^, that is, approximately 60% to 70% of the maximum rates. By contrast, Chardonnay leaves rates were depreciated by nearly 50% compared with maximum rates, but the rates were also depreciated by 50% to 66% compared with the other cultivars photosynthetic rates at 45°C.

**FIGURE 5:**
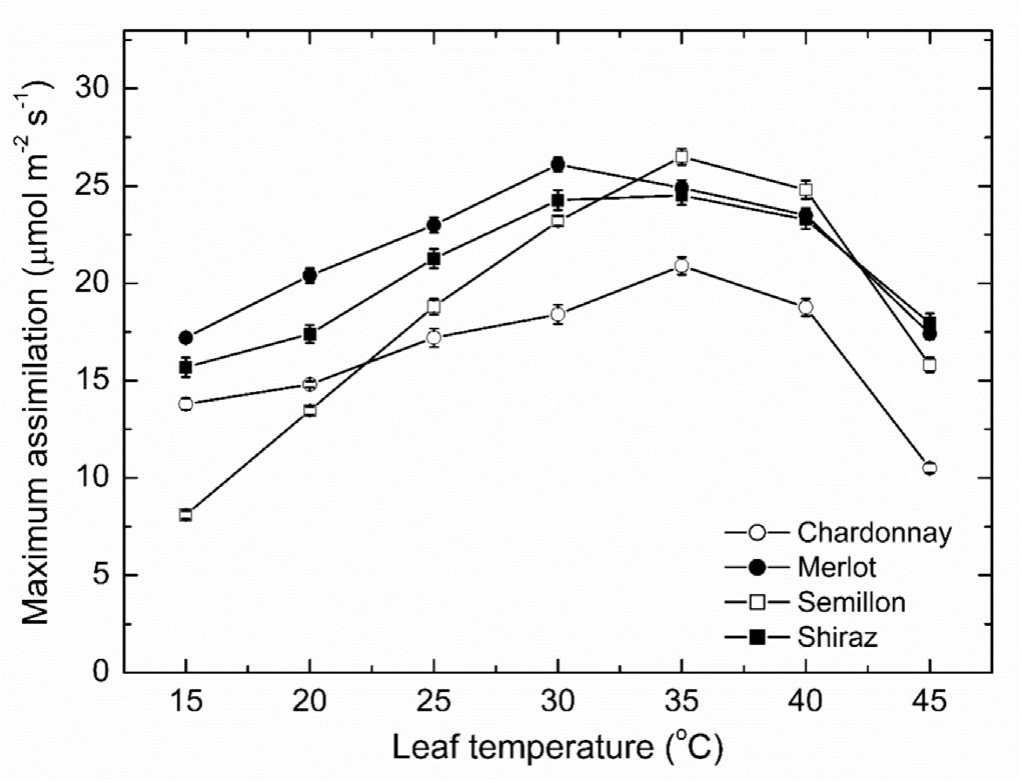
Light and CO_2_ saturated photosynthesis (mean ± SE, *N* = 4–5) as a function of leaf temperature (*A*_*max*_) for four grapevine cultivars as indicated. These data were determined as an average of the maximum rates at the highest CO_2_ concentrations.

The overall response of *A*_*max*_ to temperature (Figure 5) indicated that Merlot and Shiraz had similar temperature responses despite significant cultivar × temperature interactions (*P* < .01; *r* ^2^ = .71), both cultivars increasing rates in a near linear pattern between 15 and 30°C. Thereafter, *A*_*max*_ declined though more steeply between 40 and 45°C. Although the *A*_*max*_ rates for Chardonnay were universally lower than the other cultivars across almost all leaf temperatures, the temperature response was generally similar to that of both Merlot and Shiraz although had a slower rate of increase up to 35°C. Notably, the rates of the Chardonnay leaves declined by about .85 μmol m^−2^ s^−1^ °C^−1^ between 35 and 45°C but increased at a rate of .33 μmol m^−2^ s^−1^ °C^−1^ between 15 and 35°C. Along with the lowest CO_2_ saturated rates, the Chardonnay vines appeared to be the least tolerant of the high temperatures especially as the rates at 15°C were statistically higher compared with the rates at 45°C.

By contrast, the rates of increase in *A*_*max*_ for the Semillon leaves from 15 to 35°C increased by an average of .93 μmol m^−2^ s^−1^ °C^−1^ whereas for both Merlot and Shiraz vines, the rates of increase (15–30°C) averaged .58 and .73 μmol m^−2^ s^−1^ °C^−1^, respectively, hence slower than the rate of increase for the Semillon leaves but all faster than that for Chardonnay leaves. However, for all cultivars, the *A*_*max*_ rates decreased between 35 and 45°C in a comparable pattern, with Semillon and Chardonnay determinedly similar. Despite the apparent conformity in the curvilinear response of *A*_*max*_ to leaf temperature across the cultivars, at most temperatures, there were clear significant differences in *A*_*max*_ between the four cultivars across the whole range of leaf temperatures. Another twist in understanding the temperature responses, Merlot leaves had about the same *A*_*max*_ rates at 15 and 45°C (17 μmol m^−2^ s^−1^) whereas both Shiraz and Semillon maintained higher rates at 45°C than at 15°C, markedly so with Semillon leaves (8.1–15.8 μmol m^−2^ s^−1^). Thus, all three cultivars appeared to bias their temperature and CO_2_ dependency of assimilation towards the upper temperature range, in contrast to the Chardonnay leaves. These collective differences in *A*_*max*_, reflected in the pattern of response to leaf temperature, demonstrated the statistical cultivar × temperature variation was highly dependent on leaf temperature.

One final aspect of comparing the temperature responses of *A*_*max*_ was that in all cultivars, the optimum temperature increased in comparison with the CO_2_‐limited assimilation (Figure 2). For cvs. Chardonnay and Semillon, the shift upwards in the optimum temperature was 10°C whereas for cvs. Merlot and Shiraz, the shift was 5°C.

**FIGURE 6:**
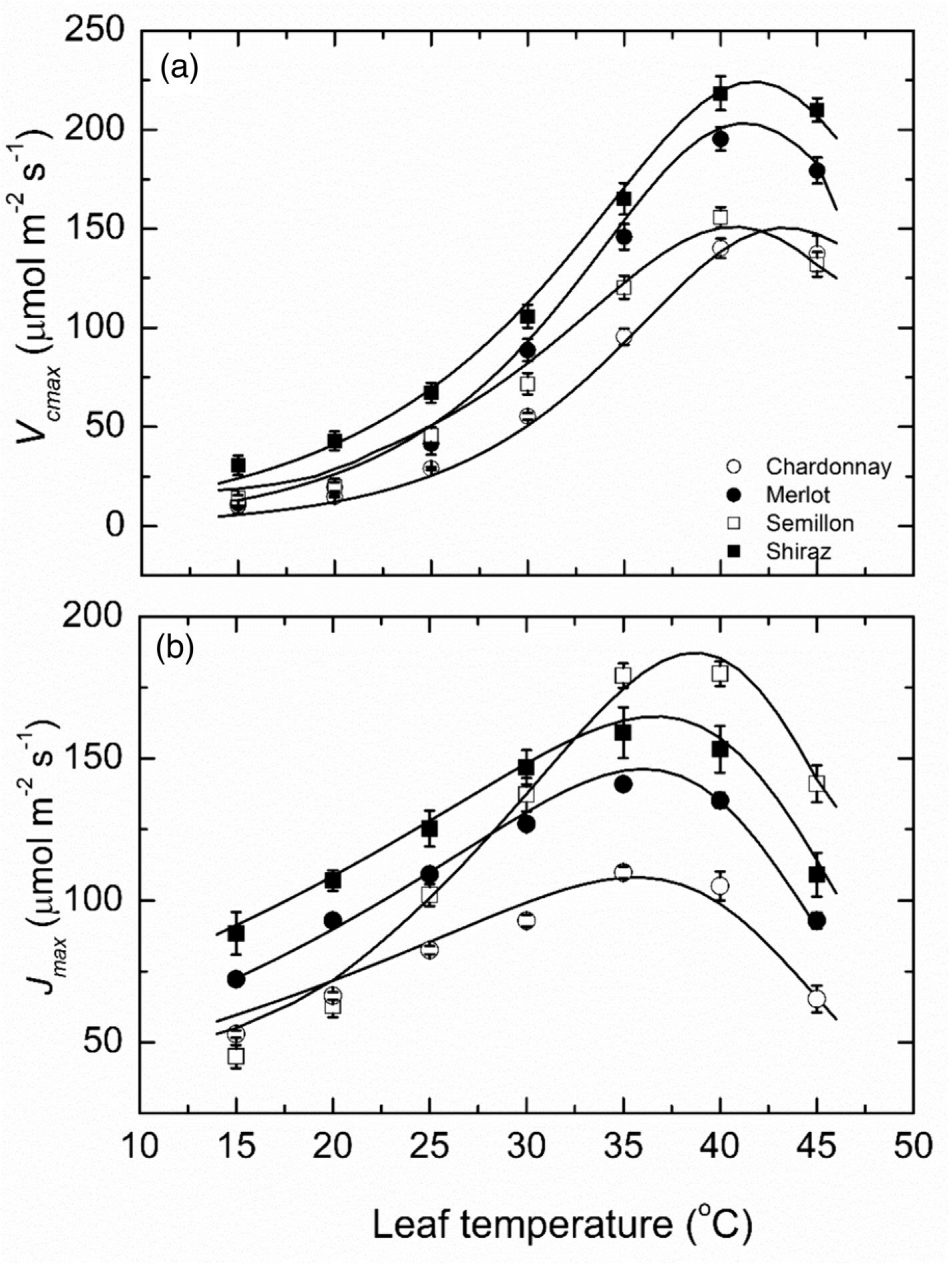
Apparent maximum rates of RuBP carboxylation (a) and regeneration (b) as a function of leaf temperature (mean ± SE, *N* = 4–5) for four grapevine cultivars as indicated. In each case, these data were derived from the C_3_ model of photosynthesis, and the lines were fitted to the modified Arrhenius function (see text).

Fitting the C_3_ model to the *A/c*_*c*_ data indicated the maximum rates of RuBP carboxylation (*V*_*cmax*_) as a function of leaf temperature for each cultivar (Figure 6a), all increased exponentially with increasing leaf temperature to maxima at either 40 or 45°C. Across Shiraz and Merlot leaves, *V*_*cmax*_ at the maximum temperature averaged about 200–215 ± 19 μmol m^−2^ s^−1^ whereas for the Semillon and Chardonnay leaves, *V*_*cmax*_ averaged about 150 ± 18 μmol m^−2^ s^−1^. Across all cultivars, some depreciation occurred in *V*_*cmax*_ at 45°C, least in Chardonnay leaves and about the same in the other cultivars. Hence, the fitting of the modified Arrhenius function with a deactivation component was highly significant (*r* ^2^ = .95–.98; *P* < .001), and at 25°C (*k*_25_), there were significant (*P* < .01) differences in *V*_*cmax*_ between the cultivars, lowest for the Chardonnay leaves at 27.9 ± 1.1 μmol m^−2^ s^−1^, 46.2 ± 2.1 to 48 ± 3.4 μmol m^−2^ s^−1^ for Merlot and Semillon and highest at 71.4 ± 4.6 μmol m^−2^ s^−1^ for the Shiraz leaves and significantly (*P* < .01) different.

The activation energy (*H*_*a*_) for the rate of increase in RuBP carboxylation with increasing leaf temperature ranged between 75.1 ± 6 and 81.4 ± 5 kJ mol^−1^ across the four cultivars, lowest for the Chardonnay leaves and highest for the Shiraz leaves and only weakly significantly (*P* < .049) different. The deactivation energy, *H*_*d*_, ranged between 201.1 and 212.5 ± 1.8 kJ mol^−1^ and was not statistically different between the cultivars. Generally, the Chardonnay leaves had the lowest *V*_*cmax*_ at almost all temperatures while the Shiraz leaves had the highest *V*_*cmax*_ across all temperatures and cultivars. Notably, cultivar differences in *V*_*cmax*_ occurred mostly above 35°C, and all cultivars were maximal at 41–43°C. There was, however, no conformity between the *V*_*cmax*_ response to temperature and the assimilation (Figures 2 and 5) response across the different cultivars.

For the most part across all cultivars, the maximum rates of RuBP regeneration (*J*_*max*_) tended to be maximal at 35 to 40°C (Figure 6b). However, the maximum rates of RuBP regeneration were significantly (*P* < .01) different between the cultivars and averaged between 180 ± 5 μmol m^−2^ s^−1^ for the Semillon leaves and 120 ± 7 μmol m^−2^ s^−1^ for the Chardonnay leaves. The fitting of the modified Arrhenius function, including a deactivation term, was highly significant (*r* ^2^ = .94–.99; *P* < .001), and at 25°C (*k*_25_), there were some cultivar differences in the maximum rates of RuBP regeneration, with *J*_*max*_ averaging 125.9 ± 6, 100 to 112 ± 5, and 75.3 ± 3 μmol m^−2^ s^−1^, for Shiraz, Semillon, and Merlot, and lowest in Chardonnay vines, respectively. Despite some of the differences in the *J*_*max*_ response to leaf temperature between the cultivars, there were few differences in the activation energies from the fitting of the modified Arrhenius function, with *H*_*a*_ ranging from 28.2 ± 2.9 to 31.6 ± 2.8 kJ mol^−1^ in Chardonnay, Merlot, and Shiraz vines, and reflecting the conformity in the patterns of change in *J*_*max*_ with increasing temperature. However, for Semillon, *H*_*a*_ averaged 57.6 ± 6.8 kJ mol^−1^, nearly twice as high and significantly different and most probably reflected the steepest increase in *J*_*max*_ between 15 and 35°C (2.36‐fold) for this cultivar. By contrast, the deactivation component was not significant and averaged 199–205 ± 5 kJ mol^−1^ across the cultivars. Nevertheless, there were marked and significant cultivar differences in *J*_*max*_ at 45°C, with Chardonnay leaves lowest at 65 ± 8.5 μmol m^−2^ s^−1^ and highest in the Semillon leaves at 140 ± 12 μmol m^−2^ s^−1^. In addition, there were marked differences in the proportional decrease in *J*_*max*_ from 35–40°C to 45°C, declining by 30% for Shiraz and 42% and 50% for Merlot and Chardonnay leaves, respectively. Notably, the *J*_*max*_ response to leaf temperature for these cultivars was closely comparable with the response of *A*_*max*_ (Figure 5), particularly the effect of the higher temperatures discriminating between the cultivars.

## 4 DISCUSSION

### 4.1 General discussion

The comparison of four grapevine cultivars growing in common conditions and the response of physiological responses to temperature has revealed inherent genotypic cultivar differences were apparent, hence conforming to the hypothesis, especially in terms of tolerance or sensitivity to the high‐temperature conditions. In all cases, these cultivars have experienced exposure to temperatures ranging between 35 and 45°C as a normal part of the growing season conditions. For cv. Semillon, it has been established (Greer & Weedon, 2016) that vegetative and reproductive growth processes had an upper threshold temperature of 35°C and any exposures above that were deleterious for growth. As examples, leaf expansion, stem extension, and rates of sugar accumulation were optimal at 30°C and particularly sensitive to exposures at 35°C and higher. Elsewhere (Aljibury et al., 1975), for cvs. Semillon, Chardonnay, and Chenin Blanc, shoot growth was reduced and berry growth delayed when a threshold of 32°C was exceeded. Similarly, shoot dry weight growth of Shiraz, Riesling, and Sultana vines were detrimentally affected above 30°C while stem extension was impaired above 35°C (Buttrose, 1969). Furthermore, exposure of Semillon vines to 40°C for 4 days at the mid‐ripening stage caused delayed sugar accumulation and severely reduced leaf photosynthesis (Greer & Weston, 2010b). For this study, 10 mg carbon g (berry dry weight)^−1^ day^−1^ was required for berry sugar to accumulate but the heat exposure reduced photosynthesis to below 4 mg carbon g (leaf dry weight)^−1^ day^−1^, and these authors concluded that rate was inadequate for berry sugar accumulation; hence, a delay in ripening occurred. Thus, the link between the impacts of high‐temperature stress on photosynthesis and those impacts on vine growth occurred through the carbon supply and demand.

### 4.2 Cultivar differences in the light responses at different temperatures

For photosynthesis at ambient CO_2_ conditions, there was apparent genotypic variation between the four cultivars in response to temperature. In all cases, rates of assimilation in response to leaf temperature of the cultivars were completely different. First, cv. Semillon vines clearly maintained the highest light‐saturated assimilation rates and cv. Chardonnay vines had the lowest rates, almost universally over all leaf temperatures. There was nearly 5 μmol m^−2^ s^−1^ difference between Shiraz and Chardonnay photosynthesis at the optimum temperatures, nearly twice that determined by Rogiers et al. (2009), and a similar difference between Semillon and the other cultivars. By contrast, Tomás et al. (2014) compared photosynthesis of seven different grapevine cultivars and the highest rates (13–22 μmol m^−2^ s^−1^) only matched those for Semillon; however, growth and measurement temperatures were not provided in that study.

Second, the pattern of response to temperature differed, with optima between 25 and 30°C for most cultivars; however, for cvs. Chardonnay and Merlot vines, the optima erred between 20 and 25°C. Semillon vines, by contrast, had a steep temperature response with marked optima at 25°C and about equal deprecation in assimilation with increasing and decreasing leaf temperatures about the optima. For cv. Shiraz, there was also a relatively steep response to increasing temperatures, but this cultivar maintained the broadest optima between 30 and 40°C. While there were similar temperature patterns for Chardonnay and Merlot vines, especially the strong deprecation in assimilation that occurred at the higher temperatures above 25°C, but there were still differences in the temperature response, especially at lower temperatures. These different assimilation responses to temperature between the cultivars were a strong indication of inherent genotypic variation and consistent with the hypothesis.

Third, photosynthetic rates at 45°C varied significantly between the cultivars, with Shiraz highest at 7.5 μmol m^−2^ s^−1^, Merlot and Semillon at about half that rate, and Chardonnay vines lowest at about a third of the Shiraz rate. These differences may have been related to genotypic differences in high rates of photorespiration at these temperatures (Greer, 2015b; Zhang et al., 2013) between the cultivars.

Although the differences were not large, the photosynthetic rates at 15°C for Chardonnay, Semillon, and Shiraz were the lowest and were markedly higher for Merlot. A further characterization of the temperature response was that Chardonnay especially, but Semillon and Merlot maintained higher ambient rates at 15°C compared with 45°C while Shiraz maintained higher photosynthetic rates at 45°C compared with 15°C. These attributes would suggest the Merlot would appear to be most tolerant of the high temperatures. However, these cultivar differences in photosynthetic rates were partially at odds with Rogiers et al. (2009), where Semillon had the highest rates, then Chardonnay and Merlot, and Shiraz with the lowest rates but both studies confirm the genotypic differences in assimilation. Although the growth or measurement conditions were not provided in the Rogiers et al. (2009) study, perhaps these may have contributed to the differences in cultivar ranking between the two studies. Other grapevine studies with different species and cultivars also have demonstrated severe impacts of 40–45°C leaf temperatures on photosynthesis, including *Vitis amurensis* (Luo et al., 2011), *V. vinifera* cv. Hongti (Xiao et al., 2017), cv. Pinot Noir (Frioni et al., 2019), and cv. Riesling (Schultz, 2003). From the present study and these citations, it remains apparent that inherent genotypic differences occurred in the temperature‐dependent photosynthetic process, especially at high temperatures. However, there are not many cultivars that stand out as potentially tolerant of the changing climate with the exception of Shiraz.

Further genotypic differences of the present study were somewhat apparent when the attributes of photosynthetic response to PFD was examined. The light limited response, the efficiency of light capture, that of the photon yield of photosynthesis, to temperature was largely similar across all cultivars, increasing most steeply from low to optimal temperatures and then declining progressively as leaf temperatures increased. Some cultivar differences were apparent at selected leaf temperatures, however. For example, the efficiency of light capture of Semillon vines was highest at the low (<25°C) range where the efficiency of Chardonnay vines also tended towards the lowest efficiency. At the highest temperature, Merlot vines had the highest efficiency and Semillon had the lowest. A similar photon yield response to temperature occurred with vegetative Shiraz vines, with efficiency declining from the highest photon yield at 20°C to the lowest at 45°C (see also Greer, 2019b; Schultz, 2003) hence comparable with the present results. For cv. Sangiovese vines (Cartechini & Palliotti, 1995), the photon yields at 25–27°C were within the same range as in the present study. The low temperature‐induced reduction in efficiency of light capture was consistent with some literature. For example, a reduction in photon yields occurred with Braestar apple trees (Pretorius & Wand, 2003) and Red Gala apple trees (Greer, 2018c). By contrast, the photon yields are more generally known to increase with increasing temperature (Gardiner & Krauss, 2001; Gindaba & Wand, 2007; Greer & Halligan, 2001); hence, the responses of these grapevine cultivars appeared to be unusual in having a declining photon yield at high temperatures, again perhaps, an effect of the high‐temperature growth regime. Notably, for all grapevine cultivars, the photon yield temperature response broadly conformed to the overall response of photosynthesis to temperature, except that cultivar differences in photon yield were relatively small whereas the assimilation rates varied markedly between the cultivars and interacted particularly with leaf temperature.

Yet further genotypic contrasts in temperature response between the cultivars were apparent when the PFD saturating photosynthesis was compared. Light saturation of Chardonnay and Merlot were only slightly responsive to temperature, with saturation mostly between 700 and 900 μmol (photons) m^−2^ s^−1^ across all temperatures. Nevertheless, light saturation of assimilation was generally lower in the Chardonnay vines compared with Merlot vines, but both tended to decrease across the higher temperature range. By contrast, PFD saturation for both Semillon and Shiraz vines were strongly temperature dependent, increasing from the lowest to an optimum temperature of 35°C for both cultivars. Notably, both cultivars saturated photosynthesis at 15–20°C at about 400 μmol (photons) m^−2^ s^−1^ but Shiraz increased to about 700 μmol (photons) m^−2^ s^−1^ at 35°C and consistent with Chardonnay, whereas the Semillon vines increased saturation to greater than 1200 μmol (photons) m^−2^ s^−1^ at 35°C leading to the conclusion that assimilation of Semillon vines was a high light demanding cultivar. However, most cultivars were comparable in that there were no marked changes in the saturating PFD at high temperatures, in contrast to the major decreases in assimilation at 40 and 45°C in all cultivars. It maybe that high light saturation for Shiraz and Semillon was required at the high temperatures to counteract the high levels of photorespiration to maintain some positive assimilation. Once again, therefore, Shiraz and perhaps Semillon were the cultivars most tolerant of the high temperatures and bode well for the future climate. Nevertheless, across all cultivars, there were genotypic differences in the PFD required to saturate photosynthesis across the different leaf temperatures. By comparison, photosynthetic saturation of cold‐grown Cox's orange apple trees required greater than 1400 μmol (photons) m^−2^ s^−1^ to saturate assimilation at 20°C but declined to about of that at 32°C (Greer, 2015a), and a similar response occurred with warm‐grown cv. Red Gala apple leaves (Greer, 2015b). However, for Braestar apple leaves (Pretorius & Wand, 2003), light saturation apparently increased from about to similar high saturation between 20 and 35°C. Similar increases in light saturation to that of Semillon also occurred with field‐grown cv. White Riesling vines from as leaf temperatures increased from 17 to 34°C (Schultz, 2003). Thus, the light saturation responses to temperature for cvs. Semillon and Shiraz were well in keeping with these and other studies (Man & Lieffers, 1997). It was notable that for three field‐grown cultivars of *Prunus persica* var. Maxima, light saturation at 25°C were comparable with Chardonnay and Merlot light saturation (Wang et al., 2007) although the growth conditions were not specified. However, these results also complied with the grapevines in that the trees expressed modest changes in light saturation as function of leaf temperature.

### 4.3 Cultivar differences in chloroplast CO_2_ responses at different leaf temperatures

The marked genotypic differences in ambient photosynthesis were also strongly apparent in the CO_2_‐saturated photosynthesis (*A*_*max*_) in response to leaf temperature. In all cases, there was an apparent curvilinear assimilation response with optimal photosynthesis at 30–35°C, but inherent genotypic variation in the maximum rates occurred as well as in the pattern of changes as leaf temperatures increased. The highest CO_2_ and light‐saturated assimilation rate occurred with Semillon (in accordance with Rogiers et al., 2009) although Shiraz and Merlot were not far behind. The comparable rate for Chardonnay was 20% lower than the other cultivars and above 25°C, the assimilation rates for Chardonnay were markedly lower than for the other three cultivars, again suggesting this cultivar was intolerant of the high temperatures. Photosynthesis (*A*_*max*_) of Semillon was again the most responsive cultivar to leaf temperature, with rates increasing from 15 to 35°C by nearly 3.5‐fold, with Shiraz, the comparable rate was 1.7‐fold increase, and for Merlot and Chardonnay at 1.5‐fold increase. Consistent with Chardonnay being the least tolerant of the high temperatures where *A*_*max*_ declined by twofold whereas *A*_*max*_ declined for the other three cultivars by 1.4–1.7‐fold. Furthermore, at 45°C, where the Chardonnay *A*_*max*_ rates were more than 60% lower than for the other three cultivars. Notably, Merlot and Shiraz maintained highest both CO_2_‐limited and CO_2_‐saturated photosynthesis at 45°C and again supportive of at least Shiraz being a high‐temperature tolerant cultivar. By contrast there were few cultivar differences in assimilation rates at the optimum temperatures of 30–35°C. However, both Shiraz and Semillon vines maintained higher CO_2_‐saturated rates at 45°C compared with 15°C although only Shiraz managed the same outcome with the CO_2_‐limited photosynthesis. Thus, the evidence that these two cultivars were biased towards the high‐temperature range conforms to be the most tolerant of the high temperatures. The evidence that Merlot was high‐temperature tolerant was not compelling, given that rates at 15 and 45°C were comparable but that this cultivar had the highest CO_2_‐limited rates at 15°C was in contradiction to high‐temperature tolerance. Perhaps a further indication of Semillon high‐temperature tolerance was apparent at 15°C where the lowest CO_2_ saturated rate occurred and perhaps an indication of low‐temperature damage occurred to assimilation. There is insufficient evidence elsewhere to suggest high‐temperature tolerance might be traded off with low‐temperature intolerance as appears to have occurred with Semillon, but it remains possible. Elsewhere, optimal photosynthesis of cv. White Riesling vines occurred at 30°C but a 1.5‐fold decrease in rates occurred at >38°C leaf temperatures (estimated from Schultz, 2003) and consistent with the current cultivars. For two other grapevine cultivars, cv. Aragonez and cv. Trincadeira, photosynthetic rates declined when exposed at 36–40°C by 2.3‐ and 3.2‐fold compared with the optima at 30°C (estimated from Costa et al., 2012) and thus most consistent with cv. Chardonnay. Similarly, for cv. Hongti, rates decreased by fivefold between 28 and 40°C (Xiao et al., 2017), and for *V. amurensis*, it declined by 2.8‐fold from 25 to 45°C (Luo et al., 2011). By contrast, for field‐grown cv. Thompson seedless vines (Kriedemann, 1968), optimal photosynthesis occurred at 30°C but a fivefold reduction in rates occurred when exposed to 45°C indicating this cultivar was far more sensitive to the high temperatures compared with all other cultivars mentioned above. There are clearly many grapevine cultivars that do not appear to be well adapted to future increasing temperatures. A notable feature of the present cultivars of elevating the source CO_2_ for assimilation, was that most cultivars shifted the photosynthetic optima upwards, by 10°C for Chardonnay and Semillon while for Shiraz and Merlot, the increase was 5°C. This suggests the marked decrease with CO_2_‐limited photosynthesis at high temperatures was probably because of photorespiration (Düring, 2003; Zhang et al., 2013) while this would have been suppressed for the CO_2_‐saturated photosynthesis and accounted for the higher rates above 35°C and also account for the upwards shift in the optimum temperatures. If ambient CO_2_ continued to increase, this may have a beneficial effect for assimilation at the probable higher temperatures.

Having established and confirmed the hypothesis that there is considerable genotypic variation between the four grapevine cultivars in the assimilation process and the leaf temperature response, then the question must be asked as to whether the hypothesis holds for those processes that underpin assimilation. Indeed, the maximum rates of RuBP carboxylation also varied between cultivars, with variation in the temperature‐induced maximal rates, highest rates occurred in cv. Shiraz vines and lowest rates occurred in cv. Chardonnay and the cultivar differences were statistically and markedly different. Across all cultivars, *V*_*cmax*_ increased according to the modified Arrhenius function with increasing temperatures up to an optimum of about 42°C (cf. Dreyer et al., 2001; Wullschleger, 1993) in all cases, but 45°C was perhaps slightly detrimental, with only small differences between the cultivars. A lower optimum of 36°C was reported for Riesling vines (see also Düring, 2003; Schultz, 2003). There is some evidence that Rubisco, the activation state, and Rubisco activase are inhibited or inactivated at these high temperatures (Hozain et al., 2010; Law & Crafts‐Brandner, 1999; Luo et al., 2011; Salvucci & Crafts‐Brandner, 2004), and decreased assimilation at ambient (Figure 2) and elevated CO_2_ (Figure 6) may have conformed with this conclusion. However, the decreased assimilation of all cultivars above 35°C did not conform with the effect of high temperatures on the apparent maximum rates of RuBP carboxylation, which were relatively unaffected at 40–45°C, by comparison. In part, this may have reflected the fact that the above effects on Rubisco metabolism were largely conducted on plants grown in amenable conditions rather than the extreme high temperatures that these grapevine cultivars are grown in. However, similar decreases in RuBP carboxylation above 40°C leaf temperature occurred with cool‐grown White Riesling grapevines (Schultz, 2003), but the rates of carboxylation were much lower than occurred here with the present cultivars. By comparison, rates at 25°C for cv. Syrah (Prieto et al., 2012) over the growing season were within the range reported for the present cultivars but lower than for Cabernet Sauvignon vines (Iacono et al., 1995) and hence generally consistent with other grapevine cultivars. Although not measured at high temperatures, *Quercus douglasii* (Xu & Baldocchi, 2003) experienced similar high growth temperatures (>40°C) as the present study and rates of RuBP carboxylation (at 25°C) in mid‐season were well comparable with the grapevine rates. Furthermore, temperate tree species (*Acer*, *Betula*, *Fagus*, *Fraxinus*, and *Quercus species*) grown at warm conditions (mean air 22°C) had rates of RuBP carboxylation at 40°C ranging from 140 to 225 μmol m^−2^ s^−1^ (Dreyer et al., 2001), hence in keeping with the present study. Thus, the grapevines grown in a hot climate in common with other species were clearly able to maintain high rates of RuBP carboxylation at high temperatures, even though the maximum rates at 40–45°C varied between the cultivars.

The fitting of the modified Arrhenius function to RuBP carboxylation, which measured the temperature‐dependent rate of increase in *V*_*cmax*_, has enabled a cultivar comparison of RuBP carboxylation sensitivity to increasing temperatures. This indicated relatively small differences occurred with slightly higher activation energy for Shiraz and Merlot compared with Chardonnay and Semillon, but overall, there was no evidence to suggest the RuBP carboxylation response to temperature was intrinsically different between the cultivars. The activation energies of the grapevine cultivars averaged between 75 and 81.5 kJ mol^−1^, and these were well within the range reported for deciduous woody plants reviewed by Kattge and Knorr (2007) and Medlyn, Dreyer, et al. (2002), but notably all species were grown at much cooler conditions than the grapevines. It was apparent, however, that *V*_*cmax*_ and *H*_*a*_ can vary seasonally as shown for Shiraz vines, where *V*_*cmax*_ declined while *H*_*a*_ increased as the growing season progressed (Greer, 2019b). Seasonal changes also occurred in two provenances of maritime pine (*Pinus pinaster*) trees (Medlyn, Loustau, & Delzon, 2002) although *V*_*cmax*_ had only minor seasonal changes while *H*_*a*_ tended to be high in midwinter and low in spring. However, *H*_*a*_ was much higher for the grapevines compared with the pine trees (47–68 kJ mol^−1^) suggesting RuBP carboxylation was more responsive to the range of temperatures compared with the maritime, cool‐grown pine trees. By contrast, several *Eucalyptus* species had seasonal increases in *V*_*cmax*_ (Lin et al., 2013) with much higher rates of RuBP carboxylation in summer than for the grapevines. However, *H*_*a*_ for the *Eucalyptus* trees was lower (59.9 kJ mol^−1^) than for the grapevines, despite both being grown in a hot but coastal (trees) climate compared with inland (vines). In addition, for cv. Cox's orange apple trees grown in a cold climate, the activation energy was shown to be leaf nitrogen dependent (Greer, 2018b) but *H*_*a*_ for well‐nourished trees averaged 107.4 kJ mol^−1^ and perhaps indicative of the cold climate enhancing temperature sensitivity. Consistent with this, hot grown cv. Red Gala apple trees averaged *H*_*a*_ over the growing season between 60 and 80 kJ mol^−1^ (Greer, 2015b). While for cool‐grown temperate tree species, *H*_*a*_ averaged 73 kJ mol^−1^ (see also Dreyer et al., 2001), thus conforming with the grapevines grown in as hotter climate. In conclusion, RuBP carboxylation varied genotypically across the grapevine cultivars and conformed with the hypothesis. However, the strong cultivar × temperature interaction that was evident for assimilation was not supported by the temperature dependency of RuBP carboxylation.

However, rates of RuBP regeneration (*J*_*max*_) a in response to the leaf temperatures were generally similar between the cultivars, however, there were also marked cultivar differences. For cv. Chardonnay and Merlot vines, *J*_*max*_ was broadly optimal at 35°C (see Dreyer et al., 2001; Wullschleger, 1993) and rates increased in concert with rising leaf temperatures and were depreciated as the leaf temperature increased above the optimum. A similar temperature response occurred with *J*_*max*_ for cvs. Semillon and Shiraz vines, but both had higher optimal temperatures of 37 to 40°C, but the decrease in rates at 45°C was similar to the other cultivars. There were however, marked cultivar differences in the maximum rates, lowest for cv. Chardonnay vines at 127.5 ± 10 μmol m^−2^ s^−1^ and highest in cvs. Semillon and Shiraz vines at 180.3 ± 12 μmol m^−2^ s^−1^, and the differences were significant. Notably, the depreciation in RuBP regeneration rates at 45°C was proportionally much greater than for RuBP carboxylation, suggesting electron transport was detrimentally affected compared with CO_2_ fixation at the supra‐high temperatures. However, similar contrasts occurred between *V*_*cmax*_ and *J*_*max*_ for cold‐grown apple trees but at 30–35°C (Greer, 2018b). For White Riesling vines, RuBP regeneration rates were in the same range and optimal at 30°C (Schultz, 2003), thus broadly consistent with cv. Chardonnay. However, RuBP regeneration of the Riesling vines declined markedly as leaf temperatures increased from 28–42°C to about half of the Chardonnay vines, thus significantly lower compared with the present cultivars at a comparable temperature. For field‐grown Syrah vines, *J*_*max*_ at 27°C from flowering to harvest were at a similar range to those rates for Merlot vines (Prieto et al., 2012). However, *J*_*max*_ for two Portuguese grapevine cultivars, Tinta Amarela and Periquita, at 28°C were well below the *J*_*max*_ rates of all the cultivars here, perhaps indicative of growth regimes differences. Furthermore, for a range of *Eucalyptus* species growing in a comparable hot climate, rates of RuBP regeneration at 25°C varied over the growing season, but there was no consistent seasonal effect (Lin et al., 2013), but the rates of RuBP regeneration were well higher than the grapevines. By contrast, *Q. douglasii* trees also growing in a hot climate (see also Wullschleger, 1993; Xu & Baldocchi, 2003) had highest rates of RuBP regeneration in spring and progressive decreases occurred thereafter but the rates in summer (at 25°C) were well consistent with those in the present study. The marked depreciation in grapevine assimilation at the high temperatures (Figures 2 and 5) were more closely associated with depreciation in RuBP regeneration than RuBP carboxylation. It was notable that the rates of RuBP regeneration for Shiraz and Semillon at high temperatures were consistent with the higher assimilation rates of these two cultivars at the high temperatures. Hence, some genotypic variation in grapevine assimilation can be ascribed with genotypic variation in RuBP regeneration.

Based on the activation energies (28.2–31.6 kJ mol^−1^) which were very similar between cvs. Chardonnay, Merlot, and Shiraz suggesting therefore, that there was no intrinsic differences in the RuBP regeneration temperature response between the cultivars. However, as the activation energy for *J*_*max*_ for the Semillon vines was nearly twice as high compared with the other cultivars, indicating the rate of increase of RuBP regeneration of cv. Semillon vines was much higher compared with the other cultivars. This was further indication of high tolerance to the growth conditions for this cultivar. For *P. pinaster* trees grown in a cool climate (Medlyn, Dreyer, et al., 2002), *H*_*a*_ varied seasonally but was consistently greater (31.4–44.5 kJ mol^−1^) than for the three main cultivars. However, the higher activation energy for Semillon is well within the range reported by the review of Medlyn, Dreyer, et al. (2002). In addition, the activation energies for various *Eucalyptus* species grown in a hot climate were comparable with that for the three main grapevine cultivars (Lin et al., 2013); however, this study failed to observe or report any interspecific differences in activation energy. Furthermore, the activation energies reported here for the grapevines were at the very bottom of the range in the Kattge and Knorr (2007) survey as well as the average of 50 ± 2.4 kJ mol^−1^, except for Semillon. Additionally, this survey revealed that no study had included growth conditions comparable with those of the present study.

## 5 CONCLUSIONS

For all photosynthetic attributes, there were clear genotypic differences, consistent with the hypothesis, and the most consistent conclusion was that cv. Chardonnay had the least tolerance to high temperatures and more generally had much lower rates of assimilation, RuBP carboxylation, and regeneration over the full range of leaf temperatures. Even at the lowest leaf temperature, Chardonnay had no assimilation advantage in comparison with the other cultivars. Notably, this was the only cultivar to maintain higher assimilation at low than at high temperatures and apparently poorly adapted to the current climatically driven high‐temperature growth conditions. Although assimilation of the Merlot vines was comparable with the other cultivars at high temperatures and had high rates of RuBP carboxylation, this cultivar uniquely had the highest rates of assimilation at low temperatures, which negates a conclusion of high‐temperature tolerance. In terms of assimilation, both at ambient and saturated CO_2_, cvs. Shiraz and Semillon maintained the highest rates but also Shiraz maintained the highest rates of RuBP carboxylation and Semillon maintained the highest rates of RuBP regeneration at high temperatures. Both these cultivars also maintained higher rates of assimilation at 45°C than at 15°C. These attributes were clearly an adaptation for these cultivars to tolerate the high temperatures that are increasingly occurring in Australian vineyards.

## AUTHOR CONTRIBUTIONS

Dennis Greer conceived the study, undertook all the data acquisition, analyzed all the data, prepared the manuscript, and takes responsibility for the integrity of the work.

## CONFLICT OF INTEREST STATEMENT

There are no conflicts of interest associated with this manuscript.

## PEER REVIEW

The peer review history for this article is available in the supporting information for this article.

## Supporting information


**Data S1.** Peer Review

## Data Availability

The data that support this study will be shared upon reasonable request to the corresponding author.
