# Supplementary material for: Intraspecific differences in the photosynthetic responses to chloroplast CO2 and photon flux density at different leaf temperatures of four grapevine cultivars grown in common outdoor conditions
Source: Plant Direct. 2024 Jun 5;8(6):e595. doi: 10.1002/pld3.595 (PMC11154808; doi:10.1002/pld3.595)
Supplement: Supplementary file 1 — Data S1. Peer Review [file PLD3-8-e595-s001.pdf]

Plant Direct

Open Access

Intraspecific differences in the photosynthetic responses to chloroplast CO_2 and photon flux density at different leaf temperatures of four grapevine cultivars grown in common outdoor conditions.

Dennis H. Greer[1]

Decision Letter Round 1:

September 28, 2023

Dr. Dennis H. Greer
Charles Sturt University
School of Ag and Wine Sciences
Wagga Wagga NSW, N/A 2678
Australia

RE: Intraspecific differences in photosynthetic responses to chloroplast CO2 and photon flux density at different leaf temperatures of four grapevine cultivars grown in common outdoor conditions

Dear Dr. Greer:

Thank you for submitting to Plant Direct. All required reviews have been returned and we have now finished our evaluation of your manuscript. In light of the reviewers' and editor's comments, further revisions are needed before the paper can be accepted for publication in Plant Direct.

Please view the editors' and reviewers' comments below and use their suggestions as a guide while you work on your revision.

When uploading the revised version of this article, please be sure to include the following:

-A word document that contains your response to the reviewers. You should respond to each reviewer comment and note the changes made to the manuscript. If you do not agree with a reviewer's comment and choose not to make a suggested revision, please explain why. Please try to provide as complete an answer as possible to each reviewer's criticisms in the "response

to reviewers" section.
-A tracked changes document
- A clean version of the latest version of the manuscript

Particularly, I would like to highlight adding/clarifying the objectives and hypothesis of the study in the introduction and ensuring the discussion links clearly to the results. Additionally, please make sure that your references represent the broader body of woody fruit crops research worldwide.

To upload your revision, please click the link below.
https://plantdirect.msubmit.net/cgi-bin/main.plex?el=A1Lr1cd6A5oOP1I3A9ftdPCg4XPi0M5xuuNXLd8NAZ

In order to provide as timely a service as possible, we ask that your revision is resubmitted within three months after receipt of this request. If an extension is needed, please send a request, along with a brief explanation, to the editorial office at plantdirect@wiley.com .

Thank you very much for giving us an opportunity to review your work. I look forward to receiving the next version.

Sincerely,
Hannah Schneider

--- Editor comments:
Particularly, I would like to highlight adding/clarifying the objectives and hypothesis of the study in the introduction and ensuring the discussion links clearly to the results. Additionally, please make sure that your references represent the broader body of woody fruit crops research worldwide.

--- Reviewer comments:
Reviewer #1:

This manuscript presents data from an assessment of single-leaf photosynthesis of four economically important cultivars of Vitis vinifera. The author measured how various

photosynthetic traits responded to increases in foliar temperature and CO2 and show relevant differences between the studied cultivars that have implications for these cultivars under high temperatures, as is currently experienced in various winegrowing regions and as is expected to become more common due to climate change. Despite this, the data are presented here more as a survey of cultivar differences and no specific hypothesis is tested for how cultivars of different genetic parentage or evolutionary histories will perform under variable temperature and CO2 conditions, and thorough discussion of these points is missing. Further, discussion of how these cultivars differ in other physiological traits that may help inform the discussion of their suitability for a warmer climate, and also contextualize the photosynthetic responses, is missing.

Specific comments for individual sections and lines are included below:

Introduction

General notes:

The introduction does an effective job of introducing the themes discussed in the paper, but at times it delves into discussing results from specific papers too specifically, causing it to read more like a Discussion section. Lines 116 to 138 provide an example, as the paragraph is devoted to discussing differences between papers and cultivars in their photosynthetic efficiency without orienting them under a single theme that is relevant to the manuscript.

While the author states that cultivars were selected due to their economic importance, the author does not include a hypothesis in the introduction detailing how they expect the cultivars to differ, or not, and why. This is especially important and would help guide the rest of the manuscript.

Line notes:

Line 31-32 - sentence is unclear, regarding effects on Merlot leaves

Line 66 - what is "significance" referring to, in relation to most Vitis vinifera cultivars?

Line 141-147 - The objective of the study is described, but no hypotheses are provided. Does the author expect cultivars to perform better/worse under at temperatures, based on those selected for this study?

Materials and Methods

General notes:

While some details are given on when measurements were performed, more specific information is necessary. If possible, it would be informative to list dates when measurements were collected, as currently it is unclear if measurements were conducted during the same time of the season, or weeks/months apart but still during the summertime.

Please see comments below regarding the statistical analysis of some of the data. It is unclear how data for a few different variables were analyzed and how significant differences between cultivars were determined.

Line notes:

Line 153-155 - How many vines per cultivar were used in this study, and were the vines planted on rootstocks? Does the author have any information about the clonal selection for the cultivars? Were specific cultivars grown in specific sized pots?

Line 163 - Since the presence/absence of fruit can affect photosynthesis rates, do all vines have fruit?

Line 211-214 - The statistical analysis used here is unclear, and specifically whether the author tested the 'cultivar' as specific fixed effect, in addition to other effects (like temperature), when assessing specific response variables. For example, in the Results text, the author specifies that there was a significant difference between cultivars for various data: for example, see lines 366-367, and lines 374-376, where statistical differences between cultivars and between temperatures within the same cultivar are presented. Clarification on this point is needed.

Results

General notes:

The resolution of the images provided is very low for a few figures (Figures 1, 2, 4), rendering it difficult to visually assess the graphs.

As mentioned below, more clarity is needed regarding how various results were statistically analyzed. It is difficult at times to understand what the p-values and r2 values included in the

text are referring to.

Line notes:

Line 259-260 - Where the text says "...there were clear cultivar differences and the interactions between cultivar and temperature were highly significant ($P < 0.01$, $r2 = 0.78$)", what is this p-value and r2 referring to? Was GLM used to assess a 'cultivar' effect, and an interaction between cultivar and temperature (cultivar*temperature)? Or were all cultivars assessed via a single model? This is unclear, and clarity is needed regarding how this data was analyzed.

Line 281-282 - Was this additional data taken using the same 4-5 leaves mentioned earlier in the legend, or using other leaves? Were they sampled on different days?

Line 288 - See earlier comment about p-values. In this case, does this p-value refer to a means comparisons between cultivars?

Line 294 - See earlier comment about p-values.

Line 299-303 - This sentence is confusing as it is written, but a statistical interaction is mentioned while no information is provided regarding the statistical interaction or how it was tested.

Line 305 - Omit 'both', since all cultivars are mentioned.

Line 352-353 - Please mention which lines have been omitted.

Line 361-364 - This sentence is written in a confusing manner, and it's difficult to understand the main point.

Line 367 - Despite being significantly different from one another, as in a means comparison, or is this in reference to an overall cultivar effect?

Line 369-371 - This sentence is confusing as written, please reword.

Lines 374-376 - Similar to previous comments, is this statistical difference the result of a comparison between temperatures within a cultivar (Chardonnay)?

Line 390-392 - Similar to previous comments, is there an interaction being tested?

Discussion

General notes: Throughout the discussion section, there are multiple sections where results are presented again, repeating what was already presented in the Results section.

Line notes:

Line 501-504 - The use of 'just' and 'certainly' within this context is confusing, and it is unclear what they mean.

Line 532-536 - This sentence should be reworded for clarity, it is a run-on.

Line 659-660 - Eucalyptus species is repeated ("for several species")

Line 689-690 - Was the data collected at mid-season?

Conclusion

Line notes:

Line 732 - "More evidence arises for the response at the high temperatures..."-what kind of evidence and for what response?

Reviewer #2:

The article "Intraspecific differences in the photosynthetic responses to chloroplast CO2 and photon flux density at different leaf temperatures of four grapevine cultivars grown in common outdoor conditions." is well-designed and executed. The abstract is well written, but I recommend adding some numerical data for the leaf temperature. The take-home message is missing in the abstract. The objective needs to be clarified in detail. Methods and results are fine, but the discussion is fragmented. Discussion should be linked with studied attributes. I recommend to add some pictures of the grape cultivars.

Decision Letter Round 2:

Plant Direct

Open Access

May 12, 2024

Dr. Dennis H. Greer
Charles Sturt University
School of Ag and Wine Sciences
Wagga Wagga NSW, N/A 2678
Australia

MSID: 2023-01278-TWR1
MS TITLE: Intraspecific differences in photosynthetic responses to chloroplast CO2 and photon flux density at different leaf temperatures of four grapevine cultivars grown in common outdoor conditions

Dear Dr. Dennis Greer:

I am pleased to inform you that your manuscript "Intraspecific differences in photosynthetic responses to chloroplast CO2 and photon flux density at different leaf temperatures of four grapevine cultivars grown in common outdoor conditions" has been accepted for publication in Plant Direct.

Your article will appear online in the next available issue of Plant Direct. To ensure your article gets published as quickly as possible, please pay attention to the steps detailed below. We have found that most of the delays happen at this stage, especially at the payment stage, so please respond as quickly as possible when prompted.

License Agreement: Once your article has been accepted it will move to Production and undergo admin and file checking - you may receive an email with any queries we have at this stage. When all required items are received by the publisher and queries resolved, the corresponding author will receive an email from Wiley's Author Services system which will ask them to log on at https://authorservices.wiley.com/bauthor and will present them with the appropriate license for completion. Your article cannot be published until both the signed license agreement and payment of the article fee have been received.

Payment of the Open Access Article Publication Fee: All articles published in Plant Direct are fully open access: immediately and freely available to read, download and share. Plant Direct charges a publication fee to cover the publication costs. The corresponding author for this manuscript should have already received a quote from the payments team (cs-openaccess@wiley.com) with the estimated article publication fee; please email cs-openaccess@wiley.com if this has not been received. The corresponding author should log on

to the Wiley Author Services site, where the publication fee can be paid by credit card or an invoice. Pro Forma can also be requested. Payment of the publication charge must be received before the article will be published online.

Proofs: You will have the opportunity to look over your paper once more when you receive the author proofs for your article. The proofs will be with you in approximately two weeks. Please note that, in addition to publishing reviewer comments, the author's responses to review comments will also be published alongside the final version of the paper. If you would not like the author's responses to be published, please contact the editorial office at plantdirect@wiley.com .

Promotion of your article: You can help your research get the attention it deserves! Check out Wiley's free Promotion Guide for best-practice recommendations for promoting your work at www.wileyauthors.com/eeo/guide. And learn more about Wiley Editing Services which offers professional video, design, and writing services to create shareable video abstracts, infographics, conference posters, lay summaries, and research news stories for your research at www.wileyauthors.com/eeo/promotion.

Thank you again for your contribution to Plant Direct. If you have any questions, feel free to contact the editorial office at plantdirect@wiley.com .

Sincerely,

Academic Editor

Esther van der Knaap

Editor, Plant Direct

--- Editor comments

--- Reviewer comments:
Reviewer #1:

Thank you to the author for the time and efforts spent in revising this manuscript and

responding to previous comments provided by the reviewers. The edits and revisions included in this latest revision answer the concerns and suggestions that I had previously provided, as the author provided a hypothesis and clearly discusses the results within the context of the hypothesis and the cultivars' suitability to changing climatic conditions. The manuscript reads well and the discussion is thorough. At this point, no concerns remain.

Author Response:

Responses to Reviewers

Editor

In concert with the Editor's suggestions, I have included an hypothesis in introduction and have made the tenor of the hypothesis the basis of the discussion and conclusions.

I have, to the best of my knowledge, captured those relevant studies in the woody fruit crops that have investigated the high temperatures as described. As it is I have referenced relevant papers from Germany, Australia, and South Africa. However, if the editor is aware of studies, I have not been made aware of, I would be grateful to hear about these.

Reviewer 1

In the general comments, the reviewer alludes to an hypothesis and I have included one as the basis of the study and used the context of the hypothesis to guide the discussion. I have also referenced the generally understood origin of the four cultivars in France that were studied but also referred to the fact that these cultivars have grown in Australian for many decades and have well adapted to the growing conditions or not, which in the jist of the paper.

The introduction has been amended to encompass the reviewers' concerns regarding the various literature which is to present the context of genotypic variation or effects of different growth conditions.

Line notes Introduction

L31-32 amended.

L 66 amended.

L141 – 147 Hypothesis is presented, and the objective amended to accommodate the hypothesis.

Line note Materials and Methods

Days of measurements and the growing season is presented. It is emphasized that to undertake 4 cultivars and repeat each response 4 – 5 times was very intensive and fully consumed both months of January and February.

The statistical analysis and the GLM procedure have been extensively enhanced in an explanation of the model used and how it was applied. This hopefully will satisfy the reviewer as to the use of P and r^2

L153 – 155 Now more clearly stated that eight vines per cultivar were used and also stated that the vines were grown on own roots (a common phenomenon in the region of study) and no clonal knowledge of each cultivar. No specific vines were grown in the smaller pots. It was more a case of the vines originally potted in the smaller pots but later the view was to use the larger pots. Competition for the potted vines meant that some smaller pots had to be used.

L163 amended to indicate fruiting vines used.

L211-214 as indicated above, the statistical analysis was amended should be clearer. In general if the cultivar x temperature interaction was significant that that was presented. If the main cultivar effect was significant then it was presented.

Results

Graphs 1 and 4 have been revised with larger symbols the journal may well make the larger.

Hopefully P and r^2 are more clearly presented. Also L294

L259-260 Revision of the statistical model should make these concerns clearer.

L281-282 Revised text in L200 and L224-225

L305 revised

L352-353 done

L361 – 364 amended text so hopefully clearer

L367 text amended to indicate a cultivar effect.

L369-371 Sentence reworded

L374 – 376 indeed a comparison of temperature within the Chardonnay cultivar and text amended.

L390-392 Text amended to indicate an interaction was being tested.

Discussion

I have extensively reviewed the discussion to remove most of the results and used alternative means to emphasize the points

L501 – 504 amended

L532 -536 text amended

L659 – 660 Text amended

L689 – 690 text amended

L732 Text amended to make this clearer

Reviewer 2

Good to hear the reviewer believes the study was well designed and executed. The abstract has been extensively amended to include a take home message and the objectives in the Introduction has been revised with an hypothesis included. I have also attempted to revise the discussion, so it flows from the hypothesis to conclusions. I regret to say I did not take any pictures of the vines as there was a strand focus each day of collecting the data.
